# Distribution, size, shape, growth potential and extent of abdominal aortic calcified deposits predict mortality in postmenopausal women

**DOI:** 10.1186/1471-2261-10-56

**Published:** 2010-11-10

**Authors:** Mads Nielsen, Melanie Ganz, Francois Lauze, Paola C Pettersen, Marleen de Bruijne, Thomas B Clarkson, Erik B Dam, Claus Christiansen, Morten A Karsdal

**Affiliations:** 1 Department of Computer Science, University of Copenhagen, Copenhagen, Denmark; 2 Nordic Bioscience Imaging A/S, Herlev, Denmark; 3 CCBR Synarc, Ballerup, Denmark; 4 Biomedical Imaging Group Rotterdam, Department of Radiology, Erasmus MC, Rotterdam, the Netherlands; 5 Wake Forest University School of Medicine, Winston-Salem, NC, USA; 6 Nordic Bioscience A/S, Herlev, Denmark

## Abstract

**Background:**

Aortic calcification is a major risk factor for death from cardiovascular disease. We investigated the relationship between mortality and the composite markers of number, size, morphology and distribution of calcified plaques in the lumbar aorta.

**Methods:**

308 postmenopausal women aged 48-76 were followed for 8.3 ± 0.3 years, with deaths related to cardiovascular disease, cancer, or other causes being recorded. From lumbar X-rays at baseline the number (NCD), size, morphology and distribution of aortic calcification lesions were scored and combined into one Morphological Atherosclerotic Calcification Distribution (MACD) index. The hazard ratio for mortality was calculated for the MACD and for three other commonly used predictors: the EU SCORE card, the Framingham Coronary Heart Disease Risk Score (Framingham score), and the gold standard Aortic Calcification Severity score (AC24) developed from the Framingham Heart Study cohorts.

**Results:**

All four scoring systems showed increasing age, smoking, and raised triglyceride levels were the main predictors of mortality after adjustment for all other metabolic and physical parameters. The SCORE card and the Framingham score resulted in a mortality hazard ratio increase per standard deviation (HR/SD) of 1.8 (1.51-2.13) and 2.6 (1.87-3.71), respectively. Of the morphological x-ray based measures, NCD revealed a HR/SD >2 adjusted for SCORE/Framingham. The MACD index scoring the distribution, size, morphology and number of lesions revealed the best predictive power for identification of patients at risk of mortality, with a hazard ratio of 15.6 (p < 0.001) for the 10% at greatest risk of death.

**Conclusions:**

This study shows that it is not just the extent of aortic calcification that predicts risk of mortality, but also the distribution, shape and size of calcified lesions. The MACD index may provide a more sensitive predictor of mortality from aortic calcification than the commonly used AC24 and SCORE/Framingham point card systems.

## Background

Cardiovascular diseases (CVD) remain the most common cause of death in the developed world, even though vast epidemiological and interventional studies have demonstrated significant declines in CVD prevalence with adherence to a healthy lifestyle, and the identification and management of risk factors[1] . Since two thirds of women who die suddenly from CVD have no previously recognized symptoms [2], it is essential to find effective indicators of cardiovascular risk that may prompt timely intervention.

Biomarkers and biochemical markers are receiving increased attention for their potential prognostic value, and for identification of those patients in most need of intervention [3]. An extensive list of more than 200 potential CVD risk factors has been compiled [4] and multivariate analysis models, such as the EU SCORE card [5] and the Framingham Coronary Heart Disease Risk Score (Framingham score) [6], have been developed to estimate risk of CVD death. However, more information may be provided by in-depth analysis of already-established risk factors.

Recently, several interesting findings have been reported on abdominal aortic calcifications as a CVD risk factor: i) Premature parental CVD has been associated with abdominal aortic calcification [7]. ii) Abdominal aortic calcium levels were significantly related to coronary calcium levels independent of the usual risk factors [8,9]. iii) In type II diabetes patients, abdominal aortic calcification was shown to constitute an independent risk factor of clinical vascular disease [10]. iv) An increased total-to-high density lipoprotein (HDL) cholesterol ratio increased the risk of presence of aortic calcification [11]. v) Lumbar aortic calcifications in bone densitometer images have been shown to constitute an independent risk factor of CVD [12]. Hence, abdominal aortic calcification is an important risk factor for CVD.

Further investigations have indicated that it is rather the number of active lipid-laden remodelling, growing, plaques, rather than the total burden of calcified plaques, including stable plaques, that is related to cardiovascular death [13]. Also the number, distribution and size of calcified plaques have been shown to relate to mortality[14]. As the Aortic Calcification Severity score (AC24) assesses, in terms of lesions, only the extent of calcification in the aorta, we developed a broader Morphological Atherosclerotic Calcification Distribution (MACD) index specifically to score the number, length, width, shape, and distribution of abdominal aortic calcifications (AAC) found in lumbar X-rays of postmenopausal women. This index was created to further understand the composition of the plaque burden in relation to cardiovascular death. Low dose computed tomography might have been used to evaluate coronary calcifications for screening purposes [15], however its cost is a limiting factor.

We evaluated whether each risk included in the composite MACD marker persisted after correction for generalized risk assessments used in the SCORE card [16], the Framingham score [17] or individual risk factors, such as smoking, cholesterol or triglycerides levels.

## Methods

### Subjects

In 1992-93, 686 postmenopausal women living in the Copenhagen area in Denmark were recruited via a household postal survey to participate in a study addressing the role of a number of metabolic risk factors in the pathogenesis of CVD and osteoporosis [18].

Follow-up was performed after 8.5 years and information about all 95 individuals who died in the observation period was obtained from the Central Registry of the Danish Ministry of Health. All participants gave informed consent to participate in 1992-93 and the study was carried out according to the Helsinki Declaration II and the European Standards of Good Clinical Practice. Local ethics committees approved the study protocol.

### Markers

At baseline, information was collected on demographics and known risk parameters such as age, weight, height, body mass index (BMI), waist and hip circumferences, systolic and diastolic blood pressure (BP), treated hypertension, treated diabetes, smoking, regular alcohol and daily coffee consumption, and weekly fitness activity. Using a blood analyzer (Cobas Mira Plus, Roche Diagnostics Systems, Hoffman-La Roche, Basel, Switzerland), fasting glucose levels and lipid profiles, consisting of total cholesterol, triglycerides, LDL-cholesterol (LDL-C), HDL-cholesterol (HDL-C), and apolipoproteins (ApoA and ApoB), were obtained.

On basis of these measurements, the composite risk SCORE card [19] and Framingham score [20] were both calculated based on the gender, age, systolic blood pressure, total cholesterol, and smoking status; and the Framingham score also based on HDL-C.

**Figure 1:**
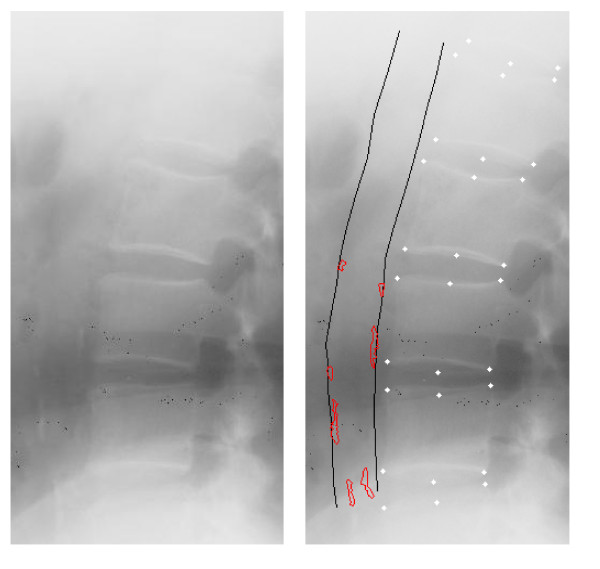
**Calcification Annotations. Lateral lumbar X-ray with calcifications in the lower region without (a) and with (b) computer-mediated annotations performed by radiologist**.

Lateral X-rays of the lumbar aorta (L1-L4 vertebrae) were taken at baseline and at follow-up. The images were digitized using a Vidar DosimetryPro Advantage scanner providing an image resolution of 9651 times 4008 pixels on a 12-bit grey scale using a pixel size of 44.6 μm^2^. Trained, blinded radiologists annotated the digitized images on a Sectra radiological reading unit using annotation software developed in Matlab (Mathworks, MA, USA) (Figure 1). The radiologists were instructed to annotate the 6 points used for vertebral height measurements on L1 - L4 [21], to delineate the aorta, and finally to outline every individual calcified deposit visible in the lumbar aorta and note their possible association to the anterior and/or posterior wall. The software enabled digital zooming and editing [22]. The inter- and intra- observer variability was tested by three radiologists annotating the same 16 randomly selected images, 3 times each.

Geometric data relating to the calcified deposits in the L1-L4 region was quantified as follows:

#### Area %

The percentage of the projected aorta lumen area occupied by calcified deposits.

#### Thickness %

The average thickness of the calcified deposits along the aorta wall, expressed as a percentage of the aorta width.

#### Wall %

The percentage of the aorta wall covered by calcified deposits.

#### Length %

The percentage of the length of the aorta in which a calcified deposit was present, in any position (anterior, posterior or internal).

#### Number of Calcified Deposits (NCD)

The number of distinct calcified deposits.

#### Simulated Plaque Area

**Figure 2:**
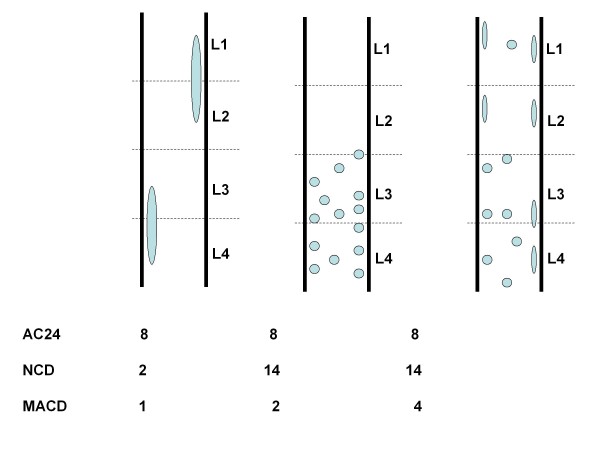
**For a given amount of calcified tissue, one can see schematically how the AC24, the NCD and the MACD can be influenced differently by variations in calcification morphology and distribution**.

As x-rays only capture the calcified core and not the biological extent of atherosclerotic lesions, we implemented a statistically validated method[14], in which the atherosclerotic plaque size was estimated from the area and form of the observed calcified lesion, and the resulting area percentage was recorded. The estimation was done using a grass-fire equation based on a morphological dilation [23] with a circular structuring element of radius 200 pixels corresponding to 8.9 mm. The biological extent of atherosclerotic lesions around an elongated calcified lesion was estimated to be larger than the biological extent of atherosclerotic lesions around a circular calcification of similar size. Thus, equal areas of calcification but of different shapes were given different scores (see Figure 2).

The relationship between these individual markers (number of plaques, their thickness, and the percentage of the aorta area, wall, and length in which plaques were detected, as well as the simulated extent of plaques) and CVD mortality in this cohort has already been demonstrated[14].

### Calculation of the MACD Index

Two novel composite markers were created:

#### 1. Morphological Atherosclerotic Distribution (MAD) factor

The Simulated Plaque Area divided by the Area estimated that portion of the biological atherosclerotic process which is not detected by x-rays.

#### 2. Morphological Atherosclerotic Calcification Distribution (MACD) index

The NCD multiplied by the MAD factor. Biologically that can be understood as the number of plaques multiplied by the disease potential score described by the MAD factor.

### Statistical analysis

Patients were stratified into survivors and deceased. The latter were sub-stratified into CVD-related, cancer-related and other-cause deaths. Since cancer and CVD have many risk factors in common, an additional group containing all cancer or CVD deaths was created to increase numbers and improve statistical significance.

### Prognostic power and additional prognostic power

To test the prognostic power, metabolic and physical parameters and AAC markers were used in separate Cox-regression models with the time of death as the outcome variable while right-censoring survivors. Significance was tested as the model weight being significantly different from zero. To test if one marker carried additional prognostic power compared to the remaining markers, a model including all elementary metabolic/physical parameters was sequentially stripped for the insignificant markers until significance persisted for all markers. To test if an AAC marker carried prognostic power in addition to the other AAC markers and/or metabolic/physical markers, each marker was compared in combined stripped models. Separate models for CVD, CVD/cancer and all-cause death were created.

### Predictive power in high risk groups

As CVD and CVD/cancer death rates were 6.5% and 15.2% respectively, a 10% percentile cut-off was used to separate subjects at high risk, from those (90%) at normal risk. Hazard ratios were computed, adjusted for the influence of other risk parameters by combining all other risk factors into Cox-regression models. Their differences were assessed by Wald tests.

### Identification of patients at risk

Finally, the power of individual markers to identify patients at risk was quantified by Receiver-Operator Characteristics (ROC) curve analysis using DeLong's test of significantly different areas under the curve (AUC)[24]. Pairs of markers, derived from pair-wise Cox regression models, were also tested to see whether identification of risk improved.

Data were expressed as mean ± standard deviation unless otherwise indicated. Variables based on concentrations, areas, or counts (including simulated area, MAD factor, and MACD index) were logged to approach normality before inclusion into models. Relative risks were computed as the per-standard-deviation increase. All tests were two-sided and considered significant when p < 0.05.

## Results

Of the 686 postmenopausal women enrolled in the original study in 1992-93, 95 died prior to follow-up with 52 (55%) of them having baseline x-ray examinations in which the full lumbar (L1-L4) aorta was visible on a single radiograph. Of these 52 deaths, 20 (38%) were due to CVD, 27 (52%) to cancer and 5 (10%) to other causes. Another 129 women had relocated from the Copenhagen area or did not want to participate in the follow-up study and provided no clinical data for it.

**Figure 3:**
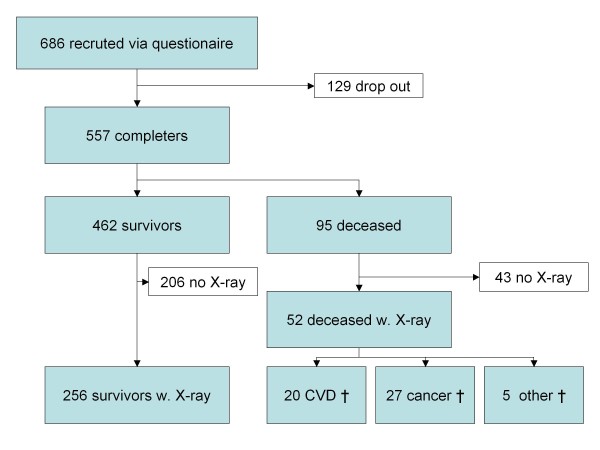
**Of 557 postmenopausal women who completed an 8.5 year follow-up study, 55% of those alive at follow-up and 55% of those who were deceased had useful X-rays with the full abdominal aorta visible in a single x-ray**. Thus, the study population included in this analysis consisted of a total of 308 women: 256 survivors and 52 deceased.

Of the 462 women completing the follow-up visit, lumbar aorta from 256 (55%) were visible on a single radiograph (Figure 3). This compares with the aorta visibility percentage reported in earlier studies [25] . Therefore in total, 308 (52 plus 256) women were included in the current analysis. Baseline demographics and risk parameters showed no difference between the discontinued women and those completing the study.

Observer reproducibility, assessed by three radiologists scoring the same 16 x-rays, three times each, resulted in both inter- and intra- observer specificity of 0.99 and an area overlap dice score of 56% and 60% respectively, showing good reproducibility[26]. These annotations were used to compute the AC24 that ranges from 0 to 24 based on the length of the vertebral sections affected by calcified deposits[27].

### Death prediction by metabolic and physical markers

Most of the physical and metabolic markers provided prognostic separation of the groups of survivors and deceased as depicted in Table 1. In a combined model including all physical/metabolic parameters only age, smoking, and triglyceride level persisted after elimination of insignificant contributions. All three parameters were positively associated with death. These were combined into one parameter denoted "combined metabolic/physical parameter" (HR per SD = 2.94 (2.18-3.95), p < 0.001) for further analysis.

**Table 1:** Population characteristics and hazard ratio of all-cause death (HR) per standard deviation of metabolic/physical markers and their 95% confidence interval based on a Cox regression model.

Physical/Systemic markers	Population (n = 308)	Survivors (n = 256)	Deceased (All cause) (n = 52)	HR per SD [95% CI] Alone	HR per SD [95% CI] Combined
Age (years)	60.3 ± 7.5	59.3 ± 7.1	65.6 ± 7.0	2.25*** (1.67-3.03)	2.41*** (1.75-3.31)

Waist (cm)	80.7 ± 10.9	80.2 ± 9.9	83.1 ± 12.4	1.29* (1.01-1.65)	-

Waist-to-hip ratio	0.80 ± 0.08	0.80 ± 0.08	0.83 ± 0.10	1.37** (1.12-1.67)	-

BMI (kg/m2)	24.7 ± 3.9	24.7 ± 3.8	25.1 ± 4.6	-	-

Smoking (%)	37	33	58	1.37** (1.08-1.73)	1.50** (1.17-1.94)

Systolic BP (mm Hg)	127 ± 21	125 ± 20	136 ± 26	1.53*** (1.20-1.94)	-

Diastolic BP (mm Hg)	77 ± 10	76 ± 10	77 ± 11	-	-

Hypertension (%)	16	15	17	-	-

Glucose (mmol/L)	5.44 ± 1.27	5.37 ± 0.99	5.79 ± 2.17	1.23* (1.03-1.46)	-

Total cholesterol (mmol/L)	6.44 ± 1.19	6.36 ± 1.14	6.85 ± 1.33	1.44** (1.12-1.86)	-

Triglyceride (mmol/L)	1.24 ± 0.75	1.15 ± 0.56	1.69 ± 1.25	1.51*** (1.29-1.76)	1.46*** (1.22-1.75)

LDL-C (mmol/L)	2.89 ± 0.82	2.85 ± 0.80	3.07 ± 0.93	-	-

HDL-C (mmol/L)	1.77 ± 0.48	1.77 ± 0.44	1.74 ± 0.62	-	-

ApoB/ApoA	0.57 ± 0.18	0.56 ± 0.17	0.64 ± 0.23	1.45** (1.14-1.83)	-

Lp(a) (mg/dL)	21.4 ± 21.7	21.9 ± 22.0	18.4 ± 19.8	-	-

EU SCORE	2.60 ± 2.58	2.16 ± 2.12	4.73 ± 3.45	1.79*** (1.51-2.13)	Not Incl.

Framingham Score	14.75 ± 3.54	14.21 ± 3.46	17.31 ± 2.74	2.63*** (1.87-3.71)	Not Incl.

Characteristics of the study population stratified into survivors and deceased (all-cause). The last column contains HR for a sequentially stripped model including all metabolic/physical markers (* p < 0.05, ** p < 0.01, *** p < 0.001).

### Death predicted by AAC markers

All imaging-based AAC markers showed higher values in the CVD, cancer, and combined CVD/cancer groups than in the survivor group (Table 2) and independently and significantly predicted death in the CVD and combined CVD/cancer groups (Table 3, column 2).

**Table 2:** Stratification of abdominal aortic calcification marker values according to cause of death shown as mean ± standard deviation.

	All (n = 308)	Survivors (n = 256)	CVD (n = 20)	Cancer (n = 27)	CVD/Can (n = 47)	Other (n = 5)	All-cause (n = 52)
**AC24**	1.67 ± 2.55	1.35 ± 2.34	3.50 ± 2.35	3.41 ± 3.23	3.45 ± 2.86	1.35 ± 2.36	3.23 ± 2.86

**Area (%)**	0.6 ± 1.2	0.5 ± 1.1	1.0 ± 0.9	1.6 ± 1.8	1.3 ± 1.5	0.5 ± 1.1	1.2 ± 1.5

**Sim. Area (%)**	11 ± 17	8.9 ± 15.7	24 ± 16	25 ± 24	25 ± 21	8.7 ± 15.5	23 ± 21

**Thickness (%)**	11 ± 20	9.0 ± l9	17 ± 16	25 ± 28	21 ± 24	8.7 ± 19	20 ± 24

**Wall (%)**	1.03 ± 1.83	0.79 ± 1.64	2.08 ± 1.70	2.51 ± 2.68	2.33 ± 2.30	0.80 ± 1.63	2.16 ± 2.27

**Length (%)**	7.5 ± 12.8	6.0 ± 11.7	15.4 ± 11.2	17.3 ± 17.6	16.5 ± 15.1	5.9 ± 11.6	15.4 ± 15.0

**NCD**	3.8 ± 7.7	2.6 ± 6.4	8.5 ± 6.5	11.6 ± 13.4	10.3 ± 11.0	2.6 ± 6.3	9.6 ± 10.8

**MAD factor**	1.50 ± 1.66	1.29 ± 1.61	3.10 ± 1.23	2.30 ± 1.46	2.64 ± 1.41	1.29 ± 1.62	2.55 ± 1.52

**MACD index**	2.19 ± 2.44	1.80 ± 2.26	4.83 ± 1.90	3.91 ± 2.51	4.30 ± 2.29	1.81 ± 1.27	4.10 ± 2.43

**Table 3:** Hazard ratio per SD increase in marker value stratified into death cause and adjusted for physical/metabolic markers, EU SCORE, and Framingham score respectively.

Adjusted by	HR/SD -	HR/SD Physical/metabolic	HR/SD EU SCORE	HR/SD Framingham
**AC24**				
CVD	1.66 (1.25-2.19)***	NS	1.38 (1.02-1.86)*	NS
CVD/cancer	1.64 (1.35-2.00)***	1.31 (1.06-1.63)*	1.40 (1.13-1.72)**	1.29 (1.02-1.63)*

**Area**				
**CVD**	1.60 (1.16-2.20)**	NS	NS	NS
**CVD/cancer**	1.68 (1.36-2.09)***	1.32 (1.04-1.66)*	1.47 (1.16-1.86)**	1.34 (1.04-1.72)*

**Sim. Area**				
CVD	2.96 (1.76-4.99)***	2.00 (1.15-3.49)*	2.46 (1.41-4.27)**	2.27 (1.26-4.09)**
CVD/cancer	2.37 (1.73-3.25)***	1.68 (1.20-2.34)**	1.96 (1.40-2.73)***	1.79 (1.26-2.54)**

**Thickness%**				
CVD	NS	NS	NS	NS
CVD/cancer	1.45(1.20-1.75)***	NS	1.27 (1.04-1.55)*	NS

**Wall %**				
CVD	1.50 (1.16-1.95)**	NS	NS	NS
CVD/cancer	1.60 (1.34-1.91)***	1.26 (1.04-1.53)*	1.42 (1.17-1.73)***	1.30 (1.05-1.62)*

**Length%**				
CVD	1.55 (1.18-2.04)**	NS	NS	NS
CVD/cancer	1.61 (1.34-1.95)***	1.26 (1.03-1.55)*	1.42 (1.16-1.73)***	1.29 (1.03-1.62)*

**NCD**				
CVD	2.44 (1.72-3.48)***	1.76 (1.20-2.60)**	2.20 (1.48-3.26)***	2.04 (1.34-3.12)***
CVD/cancer	2.28(1.79-2.90)***	1.69 (1.30-2.21)***	2.00 (1.53-2.62)***	1.86 (1.40-2.47)***

**MAD factor**				
CVD	3.37 (1.83-6.21)***	2.44 (1.22-4.89)*	3.02 (1.55-5.86)**	2.85 (1.44-5.64)**
CVD/cancer	2.19 (1.58-3.04)***	1.58 (1.11-2.26)*	1.83 (1.29-2.59)***	1.74 (1.22-2.48)**

**MACD index**				
CVD	5.22 (2.40-11.36)***	3.17 (1.48-6.78)**	4.36 (1.97-9.66)***	4.22 (1.79-9.97)***
CVD/cancer	2.99 (2.05-4.35)***	2.01 (1.37-2.95)***	2.43 (1.64-3.59)***	2.27 (1.51-3.41)***

(* p < 0.05, ** p < 0.01, *** p < 0.001)

### Additional information from AAC markers

This significance persisted for Simulated Area, NCD, MAD factor, and MACD also when adjusted for the combined metabolic/physical parameter, EU SCORE, or Framingham score. AC24, Wall%, and Length% all maintained a significant prediction under adjustment in the CVD/Cancer group, but did not have sufficient statistical power in the smaller CVD group (Table 3).

In a combined elimination model using all elementary calcification markers, only the number of calcified deposits (NCD0 (positive association to death) and Area% (negative association to death) persisted in the CVD group (HR/SD = 4.10 (2.68-6.29), p < 0.001) and the CVD/Cancer group (HR/SD = 2.80 (2.16-3.63), p < 0.001. In similar models adjusting for the combined metabolic/physical parameter, NCD persisted with positive association to death whereas Area% was substituted by Thickness% and Length% in the CVD and the CVD/Cancer groups respectively, both with a negative association to death (CVD HR per SD = 2.99 (1.84-4.87), p < 0.001, CVD/Cancer HR per SD = 2.04 (1.52-2.74), p < 0.001). The AC24 lost significant predictability when combined with the other markers and was eliminated in all combinations of patient groups and combinations with metabolic/physical markers. Simulated area was eliminated last or close to last in all elimination models. The composite marker MACD showed highest predictability in all tests and also higher predictability (but not significantly so) than the combined elimination models of the elementary calcification markers.

### Predictive power in the high-risk group

In the CVD deaths group, the highest 10% of NCD or MACD scores were significantly associated with death. This did not hold forAC24 or Area% values in the same group (Table 4). This relation persisted but with decreasing hazard ratios when adjusted by standard composite metabolic/physical markers (EU SCORE or Framingham score) or the combined metabolic/physical parameter in the elimination model from Table 1. Similar results were obtained in the CVD/cancer group with slightly lower hazard ratios and higher significance levels due to the larger population.

**Table 4:** Hazard ratio for high risk subjects based on 90% threshold in the CVD deaths group.

AC marker Adjusted by	Hazard ratio alone	AC24	Area%	NCD	MACD index
**None**	-	NS	NS	10.9 (4.4-27)***	15.6 (6.3-38)***

**EU SCORE**	4.9 (1.9-13)**	NS	NS	8.5 (3.2-23)***	13.2 (4.9-35)***

**Framingham**	NS	NS	NS	10.8 (4.1-28)***	15.7 (6.1-40)***

**All metabolic/physical**	10.1 (4.1-25)***	NS	NS	7.2 (2.8-18)***	9.8 (3.7-26)***

(* p < 0.05, ** p < 0.01, *** p < 0.001).

**Figure 4:**
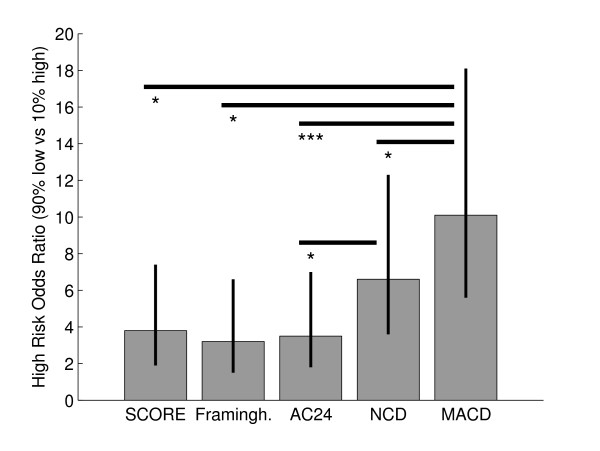
**Odds ratio of death in the CVD and cancer group who were assessed as being in the 10% at greatest risk of mortality, versus survival in the remaining subjects**. Significance of difference is indicated as * for p < 0.05, ** for p < 0.01, *** for p < 0.001 using the likelihood ratio of appropriately combined logistic regression models.

Comparing odds ratios (OR), the NCD exhibited a significantly higher OR than the AC24 score (p = 0.04). The OR for the MACD index was significantly higher than for any other marker (compared with EU SCORE, Framingham score and NCD, p < 0.05; compared with all others, p < 0.001) (Figure 4).

### Identification of patients at risk

Several markers significantly separated CVD deaths from survivors prognostically based on the area under the ROC-curve (AUC) (MACD 0.85 ± 0.06, SCORE 0.80 ± 0.06, Framingham 0.73 ± 0.07, triglyceride 0.74 ± 0.08, total cholesterol 0.77 ± 0.06) or AAC-markers (AC24 0.79 ± 0.06, Area% 0.76 ± 0.06, NCD 0.82 ± 0.07). Comparing MACD AUC to the remaining markers, DeLong's test of significantly higher AUC yielded: SCORE p = 0.50, Framingham p = 0.12, triglycerides p = 0.24, total cholesterol p = 0.16, AC24 p = 0.03, Area% p = 0.009 and NCD p = 0.14.

Combination of the MACD index with metabolic and physical markers resulted in an AUC of up to 0.89 ± 0.06 when combined with triglyceride concentrations. This combination provided the largest improvement over MACD in the low risk range and was higher than any of the other scores and significantly so (p < 0.05) except for the SCORE (p = 0.11) and NCD (p = 0.07).

## Discussion

We investigated whether more information could be obtained from calcified deposits in the abdominal aorta to better predict CVD death than the gold standard AC24, which was developed from the Framingham Heart Study cohorts. We hypothesised that the presence of many small, spatially distributed, radiographically visible calcified deposits of varying shape in the lumbar aorta had a stronger relation to CVD death than the AC24 segment-wise scoring of the extent of calcified deposits on the aortic wall.

The AC24 score [28] quantifies the burden of calcified plaques in the aorta by segment-wise scoring of the extent of calcified deposit coverage of the aortic wall. We investigated whether additional aspects of the outline of the individual plaques may be associated with the progression and/or prognosis of atherosclerosis. We analyzed the area, thickness, wall and length % of the abdominal aorta covered by calcification and the number of distinct calcified deposits. Furthermore, we calculated the simulated plaque area in which the atherosclerotic plaque size was estimated from the area and form of the observed calcified lesion. Lastly, two composite markers were created: i) The Morphological Atherosclerotic Distribution (MAD) factor was constructed by dividing the simulated plaque area with the absolute plaque area. ii) The Morphological Atherosclerotic Calcification Distribution (MACD) index given by the NCD multiplied by the MAD factor. 	In the present cohort, eight different markers (AC24, area, simulated area, wall%, length%, NCD, MAD and MACD) exhibited a significant hazard ratio per standard deviation increase for death in the combined CVD/cancer group when adjusted for physical/metabolic markers, the EU SCORE, and the Framingham score respectively. However, only four markers (simulated area, NCD, MAD and MACD) had sufficient power in risk segregation of CVD mortality when adjusted by physical/metabolic markers, the EU SCORE and the Framingham score. The composite MAD factor showed increased sensitivity to CVD compared to cancer mortality. The reason for this may be that the MAD factor essentially scores how small and widely distributed the individual calcified plaques appear. When the MAD factor was combined with the number of calcified plaques, which as an individual parameter alone was shown to be a strong predictor of mortality, the resulting MACD index displayed superior predictive power over any other marker. The MACD index produced hazard ratios >4 per standard deviation increase, even after adjustment for metabolic/physical factors. This is, to our knowledge, the strongest predictor yet of mortality based on simple x-rays.

In trying to identify which tool would be most useful in clinical practice to identify CVD patients at highest risk of death, we found, from applying the various scoring systems to postmenopausal subjects who had died from CVD, that the MACD index is potentially a better predictor of mortality. The MACD index produced a hazard ratio for death of more than 10, while the hazards ratios for the AC24 and the Framingham score were both insignificant, and the EU SCORE, had a value of the hazard ratio of 5. Based on our study, postmenopausal women identified by the MACD index as being among the 10% at greatest risk of mortality from CVD, would have a two-third probability of dying within the following 8.5 years.

## Conclusion

Atherosclerosis is a systemic disease in which lumbar aortic calcifications occur (5). Recently, increasing attention has been devoted to the correlation between the number of lumbar aortic calcifications in radiographs and coronary calcifications [29] quantified by more advanced and invasive imaging techniques such as electron beam tomography (EBT) for coronary artery calcium scoring (CACS). The publications suggest radiographs provide equally valuable information on CVD and offer the advantage of simplicity for in-office quantification [30-33]. Some studies even suggest the number of lumbar aortic calcifications is an independent predictor of CVD events [34]. Importantly, only the calcified core of an atherosclerotic lesion is detected in x-rays whereas the surrounding necrotic tissue and region of high remodelling and fibrosis are not detectable. Hence, the actual pathologically involved area is underestimated in radiographs. Consequently, the morphological enlargement of plaques (used in the MAD factor and thereby the MACD index) may carry information related to the projected area of the inflammatory processes and indirectly indicate an increased risk. This additional information may result in a better prediction of mortality risk than the current state-of-the-art, the AC24 radiographic scoring of atherosclerotic plaques. The present study has its limitations. Its findings are only valid for a follow-up period of 8.5 years and may not necessarily apply to shorter follow-ups. For short follow-up times, the predictive power could possibly be based only on the total plaque burden as described by the AC24 score. Furthermore, the present population is restricted in size, geographical and ethnic content to postmenopausal Danish women. The present study needs validation in other populations and longer term clinical settings.

In conclusion, assessment of the shape, size, number, distribution, and extent of lumbar aortic calcifications may aid in identifying patients at risk of CVD death and thus most in need of treatment.

## Competing interests

MN has received grants from Nordic Bioscience A/S and CCBR A/S. PP  has been employed by CCBR Synarc A/S. FL, MdB, EBD, and MG have been or are employed by Nordic Bioscience and Nordic Bioscience Imaging A/S. MK and CC hold shares in Nordic Bioscience A/S and CC holds shares in CCBR Synarc A/S. Patents covering parts of the methodology in this manuscript have been filed (US 20090204338 – 12/069894).

## Authors' contributions

MN made the main discovery, performed part of the statistical analysis, and coordinated the IT development. MG performed the statistical analysis. FL discovered and implemented the simulated area. PP conducted all radiological readings and annotations. MdB designed part of the study and the annotation tool. TBC and MK hypothesized the relation between the biological processes and imaging and made analysis of biological data. CC designed and coordinated the study. All authors contributed to writing the manuscript and have read and approved the final manuscript

## Pre-publication history

The pre-publication history for this paper can be accessed here:

http://www.biomedcentral.com/1471-2261/10/56/prepub
